# Mapping Genomic Heterogeneity in Pediatric and Adolescent–Young Adult Sarcomas: Insights from the Italian SAR-GEN2016 and SAR-GEN_ITA Prospective Multicenter Trials

**DOI:** 10.1158/2767-9764.CRC-25-0697

**Published:** 2026-04-17

**Authors:** Elisa Tirtei, Valeria Difilippo, Federico Divincenzo, Sebastian Dorin Asaftei, Nicola Ratto, Raimondo Piana, Pietro Pellegrino, Alessandra Linari, Mauro Papotti, Katia Mareschi, Caterina Parlato, Simonetta Guarrera, Saverio Minucci, Marco Rabusin, Carla Manzitti, Arcangelo Prete, Federico Mercolini, Roberto Luksch, Cristina Meazza, Antonina Parafioriti, Angela Tamburini, Luca Coccoli, Rosamaria Mura, Marco Zecca, Emanuela Palmerini, Toni Ibrahim, Serena Peirone, Linda Penolazzi, Elvira De Luna, Celeste Cagnazzo, Sabrina Bombaci, Ivana Ferrero, Alessia Giovanna Santa Banche Niclot, Camilla Francesca Proto, Manuela Spadea, Paola Quarello, Elena Marini, Katiuscia Gizzi, Beatrice Fenoglio, Virginia Livellara, Alessandro Di Gangi, Nadia Puma, Giovanna Sironi, Andrea Di Bernardo, Matteo Cereda, Franca Fagioli

**Affiliations:** 1 Paediatric Onco-Haematology Department, Regina Margherita Children’s Hospital, Turin, Italy.; 2 Department of Public Health and Paediatrics, University of Turin, Turin, Italy.; 3 Italian Institute for Genomic Medicine, c/o IRCCS, Candiolo, Italy.; 4 Candiolo Cancer Institute, FPO-IRCCS, Candiolo, Italy.; 5 Orthopaedics Oncology Department, AOU Città della Salute e della Scienza di Torino, Turin, Italy.; 6 Pathology Unit, AOU Città della Salute e della Scienza di Torino, Turin, Italy.; 7 Pathology Unit, Department of Oncology, University of Turin, Turin, Italy.; 8 Department of Oncology and Hemato-Oncology, University of Milan, Milan, Italy.; 9 Pediatric Hemato-Oncology, Institute of Maternal and Child Health IRCCS Burlo Garofolo, Trieste, Italy.; 10 U.O.C. Oncologia, IRCCS Istituto Giannina Gaslini, Genoa, Italy.; 11 Pediatric Hematology and Oncology, IRCCS Azienda Ospedaliero-Universitaria di Bologna, Bologna, Italy.; 12 Paediatric Oncology Unit, Fondazione IRCCS Istituto Nazionale dei Tumori, Milan, Italy.; 13 Pathology Unit, UOC di Anatomia Patologica ASST Pini-CTO, Milan, Italy.; 14 Department of Paediatric Haematology-Oncology, AOU Meyer IRCCS, Florence, Italy.; 15 Paediatric Onco-Haematology Unit, S. Chiara Hospital, AOUP Pisa, Pisa, Italy.; 16 Paediatric Onco-Haematology Unit, Azienda Ospedaliera Brotzu, Cagliari, Italy.; 17 Paediatric Haematology and Oncology, Fondazione IRCCS Policlinico San Matteo, Pavia, Italy.; 18 Osteoncology, Bone and Soft Tissue Sarcomas and Innovative Therapies Unit, IRCCS Istituto Ortopedico Rizzoli, Bologna, Italy.; 19 Sylvester Comprehensive Cancer Center, Miller School of Medicine, University of Miami, Miami, Florida.; 20 Department of Neurosciences, Rehabilitation, Ophthalmology, Genetics, Maternal and Child Health (DINOGMI), University of Genoa, Genoa, Italy.; 21 Health Science Interdisciplinary Center, Sant’Anna School of Advanced Studies, Pisa, Italy.; 22 Section of Genomics and Transcriptomics, Fondazione Pisana per la Scienza ONLUS, Pisa, Italy.; 23 IFOM ETS - The AIRC Institute of Molecular Oncology, Milan, Italy.

## Abstract

Sarcomas in pediatric and adolescent–young adult (AYA) populations represent rare and biologically heterogeneous tumors with complex genetic underpinnings. Genomic profiling reveals subtype-specific alterations and therapeutic targets. Such tumors still represent an unmet clinical need due to limited treatment options and poorer outcomes, especially in advanced stages. Here, we present the SAR-GEN2016 and SAR-GEN_ITA clinical trials, conducted across 12 Italian centers, which enrolled 201 patients, including 158 bone and soft-tissue sarcoma samples collected at diagnosis or relapse. Whole-exome sequencing was successfully performed on 120 tumor samples. The most representative histotypes were osteosarcoma (*n* = 53), Ewing sarcoma (*n* = 39), rhabdomyosarcoma (*n* = 13), and synovial sarcoma (*n* = 5), and the genomic analyses were mainly focused on these subtypes. Overall, our cohort showed genomic differences between subtypes, highlighting how genomic complex sarcomas and fusion-driven sarcomas are distinct entities. The genomic complex histotypes, such as osteosarcoma, were characterized by a lower tumor mutational burden (TMB) and higher copy-number variation burden with enrichment of the CN2 signature. Recurrent and metastatic Ewing sarcomas have a higher TMB compared with treatment-naïve primary tumors, along with increased intratumoral heterogeneity. Oncogenic pathway analyses revealed dysregulation of the RTK–RAS and NOTCH pathways across subtypes, particularly in metastatic and recurrent tumors. In 71 of 120 analyzed samples (59%), at least one potentially actionable genomic alteration was identified, and 16% of those patients with relapsed disease received a matched targeted therapy based on the molecular profiling results. All findings were classified as ESCAT tier II or III. Our findings support the value of integrating genomic and clinical data to accelerate translational research in rare tumors.

**Significance:**

Pediatric and AYA sarcomas are rare with poor outcomes in advanced stages and limited treatment options. Through the SAR-GEN2016 and SAR-GEN_ITA multicenter trials, we performed whole-exome sequencing on 120 tumor samples with matched normal tissue from 158 patients with bone and soft-tissue sarcoma. Our integrative genomic analysis supports the genomic stratification and precision oncology in rare pediatric sarcomas.

## Introduction

Bone and soft-tissue sarcomas are a rare and heterogeneous group of mesenchymal malignancies that account for approximately 1% of all adult cancers but represent 10% to 15% of solid tumors in pediatric, adolescent and young adult (AYA) populations (1, 2). The current standard treatments, based on combinations of surgery, chemotherapy, and radiotherapy, have improved survival for localized disease, but prognosis remains poor for patients with metastatic, relapsed, or refractory disease (3–7). The limited therapeutic options available for these patients highlight the urgent need to improve our understanding of the molecular basis of sarcoma biology, with the aim of identifying new biomarkers and therapeutic targets (8).

From a genomic perspective, sarcomas encompass a broad spectrum ranging from tumors characterized by highly complex genomes dominated by copy-number variations (CNV), such as osteosarcoma (9), to fusion-driven entities with relatively stable genomes, including Ewing sarcoma and synovial sarcoma (SS; ref. 10). Large-scale sequencing studies of pediatric and adult patient cohorts have revealed that osteosarcoma exhibits extensive chromosomal instability, recurrent copy-number gains and losses, and frequent inactivation of *TP53* and *RB1* (11). In contrast, Ewing sarcoma is predominantly fusion-driven and shows a lower mutational burden with limited recurrent somatic mutations beyond secondary alterations that are acquired as the disease progresses (11). Rhabdomyosarcoma (RMS) is an intermediate case. Embryonal RMS typically shows a more complex fusion-negative genomic profile that is enriched for alterations in the RAS pathway, whereas alveolar RMS is predominantly fusion-driven (12–14). Overall, previous studies have established these broad genomic categories. However, most available datasets remain constrained to single histotypes, are biased toward adult-enriched cohorts, or do not include longitudinal data across multiple disease stages (15, 16).

Early in the precision medicine era, sarcomas were underrepresented in genomic-guided therapeutic strategies, although patient enrollment has increased substantially in recent years (16, 17). Despite ongoing debate regarding the clinical impact of precision oncology in sarcomas (17), the identification of actionable genomic alterations, albeit rare, can significantly improve patient survival (18). Moreover, integrated genomic and transcriptomic studies are increasingly supporting refined prognostic stratification to better define the therapeutic strategies (19–22).

Here, we present two prospective clinical trials, the monocentric SAR-GEN2016 pilot study and the multicentric SAR-GEN_ITA study (NCT04621201), including 158 bone and soft-tissue sarcoma samples. We performed whole-exome sequencing (WXS) on 120 tumor samples, representing the largest Italian cohort of pediatric and AYA bone and soft-tissue sarcomas, encompassing 12 distinct subtypes. This unique dataset enabled us to investigate the genomic landscape of rare sarcoma subtypes in a real-world clinical setting, as well as explore the potential utility of molecular profiling in guiding clinical management. The present study focused on the four prevalent sarcoma subtypes of the cohort, with 53 osteosarcomas, 39 Ewing sarcomas, 13 RMSs, and 5 SSs, comparing molecular differences across subtypes and disease status. Therefore, this integrated genomic analysis enhanced the molecular characterization of pediatric and AYA sarcomas and provided useful information on potential targeted therapies.

## Materials and Methods

### Patients and biological specimens

All clinical data and biological samples were collected within the clinical trials entitled “Genomic Profile Analysis in Children, Adolescents, and Young Adults with Sarcomas – SAR-GEN_ITA” (ClinicalTrials.gov ID: NCT04621201) and the SAR-GEN2016 pilot study. Both trials were approved by the Independent Ethics Committee of A.O.U. Città della Salute e della Scienza di Torino - A.O. Ordine Mauriziano - A.S.L. Città di Torino (Turin, Italy; on November 30, 2018 and September 15, 2016, respectively) and by local ethical committees as required by Italian law. The trials were conducted in accordance with the principles of the Declaration of Helsinki and Good Clinical Practice. Patients and parents (if patients were younger than 18 years) were provided with written informed consent for the analysis and data publication.

**Figure 1.**
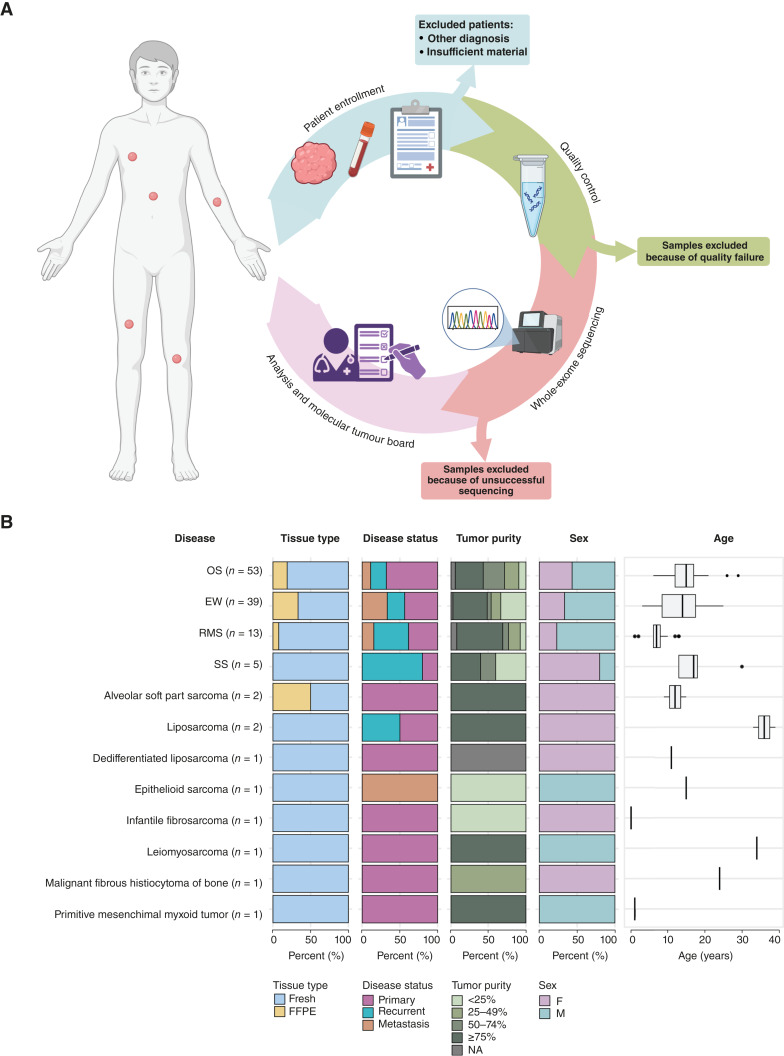
Overview of SAR-GEN2016 and SAR-GEN_ITA prospective multicentric trials. **A,** Workflow of the clinical trials, illustrating the process from patient enrollment through sample collection, genomic profiling, and analysis, to the multidisciplinary discussion at the MTB for therapeutic decision-making, with excluded samples indicated at each stage. **B,** Combination of percent stacked barcharts and box plot, from left to right: number of samples for each sarcoma subtype, tissue type of the biopsy (fresh or FFPE), disease status at the moment of the surgical procedure (primary, recurrent, and metastasis), purity of the tumor calculated after sequencing, and sex and age of the samples. EW, Ewing sarcoma; NA, not available; OS, osteosarcoma. [**A,** Created in BioRender. Grieco, M. (2025) https://BioRender.com/c7w0fbz.]

The pilot study SAR-GEN2016 was conducted at A.O.U. Città della Salute e della Scienza di Torino with the following eligibility criteria: (i) patients with a suspected first diagnosis or recurrence of bone or soft-tissue sarcoma; (ii) age <40 years; and (iii) confirmatory diagnosis validated through rigorous examination by a dedicated pathologist. The multicentric SAR-GEN_ITA trial was conducted across 12 hospitals within the National Network of the Italian Association of Pediatric Onco-Hematology (AIEOP) with the following eligibility criteria: (i) patients with a suspected first diagnosis or recurrence of osteosarcoma, Ewing sarcoma or SS; (ii) age ≤24 years; (iii) confirmatory diagnosis validated through rigorous examination by a dedicated pathologist (Fig. 1A). For each enrolled patient, biological samples were collected (tumor and healthy tissue, peripheral blood, or normal solid tissue). Both studies included nondecalcified or decalcified formalin-fixed, paraffin-embedded (FFPE) blocks with at least 20% tumor content (Fig. 1B; Supplementary Table S1). Fresh tumor samples and healthy tissue were centralized within 48 hours from collection, and they were immediately processed for genomic analysis. All procedures were performed as part of clinical practice. Clinical data and histologic features were recorded in specific Case Report forms. Clinical details collected included patients’ characteristics (sex, age, and clinical history), sampling details (anatomic site of biopsy or surgical procedure), and clinical follow-up (Fig. 1B).

### Multidisciplinary molecular tumor board

Both trials incorporated a molecular tumor board (MTB), consisting of a multidisciplinary team of experts, to systematically evaluate the genomic profiles of individual patients. The MTB reported somatic or germline genomic alterations as “potentially actionable” when the identified molecular lesion was theoretically targetable by an approved or investigational drug, either directly or indirectly within the affected pathway. To better define the clinical relevance of these targets, the European Society of Medical Oncology (ESMO) Scale for Clinical Actionability of Molecular Targets (ESCAT) was adopted (23). The ESCAT scale provides an evidence-based framework to classify and prioritize cancer genomic alterations according to their clinical actionability, supporting patient selection for targeted therapies (23). The ESCAT tiers are described in Supplementary Table S2. For each patient, a clinical report was generated, summarizing the genomic findings and, when applicable, providing therapeutic recommendations.

### Genomic DNA extraction

Genomic DNA from fresh tumor and healthy solid tissue samples was extracted using the DNeasy Blood and Tissue Kit (QIAGEN) following the manufacturer’s protocol, or the genomic DNA from the tumors was extracted from 2-μm-thick FFPE sections (8–10 sections per sample) using Maxwell RSC DNA FFPE Kit (Promega Corporation) on Maxwell RSC 48 Instrument (Promega Corporation) following the manufacturer’s protocol. DNA from peripheral blood samples was used as a matching reference and extracted using the automatic QIAcube extractor (Qiagen), following the instructions of the DNeasy Blood & Tissue Kit (Qiagen).

### WXS and sequence alignment

Whole exome was captured from genomic DNA for tumor and matched normal tissue using the SureSelectXT Human All Exon V6 + COSMIC (Agilent), following the manufacturer’s protocol as previously described (24). Sequence alignment was performed as previously described (25). Briefly, somatic and germline variants were identified according to GATK Best Practices, implemented via the HaTSPiL framework (RRID: SCR_001876; ref. 26). Sequencing reads were aligned to the GRCh37/hg19 reference genome using Novoalign (RRID: SCR_014818; http://www.novocraft.com/) with default settings, allowing up to three mismatches per read. PCR duplicates were marked with Picard’s MarkDuplicates, and local realignment around indels was performed with GATK’s RealignerTargetCreator and IndelRealigner (RRID: SCR_001876; Broad Institute, 2022).

### Identification of somatic variants and selection of cancer driver mutations

Variant calling for single-base substitutions (SBS) and small insertions/deletions (indel) was conducted independently on tumor and matched normal samples as previously described (25). Briefly, MuTect v1.1.17 (RRID: SCR_000559; ref. 27), Strelka v1.0.15 (RRID: SCR_005109; ref. 28), and VarScan2 v2.3.6 (29) were used, and only high-confidence variants (flagged as “KEEP” in MuTect and “PASS” in Strelka) were retained, with additional filters requiring an allele frequency ≥5% and a read depth ≥10×. Somatic variants were annotated using ANNOVAR and classified as nonsilent based on functional impact (RRID: SCR_012821; ref. 30). Splicing mutations were considered when located within at least 2 bp from splice sites. Cancer driver and actionable genes were prioritized, and mutations were retained based on recurrence (detected by at least two variant callers). Selected somatic mutations were subsequently summarized and visualized using the R package *maftools* v.2.24.0 (31).

### Copy-number variations and recurrency analysis

Somatic copy-number variations (CNV) were identified as previously described (25). Briefly, CNVs were called using Sequenza v3.0.0 (RRID: SCR_016662; ref. 32) with a 5 Mb window and minimum read depth of 10×. Genes overlapping at least 80% with CNV regions were either classified as amplified or deleted based on GENCODE annotations (33). Sequenza was also used to estimate tumor purity and ploidy. Samples without matched normal tissue were excluded from CNV analysis. To assess the CNV burden of each sample, the *read_copynumber* function from the *sigminer* R package v2.2.2 (34) was used, following the package instructions for Sequenza analysis (32). Recurrently amplified and deleted cytobands and genes were identified using GISTIC2 v2.0.23 (35) with the following parameters: genegistic = 1, savegene = 1, armpeel = 0, conf = 0.95, brlen = 0.5, and gcm = extreme. The readGistic function from the *maftools* R package v.2.24.0 (31) was used to manage the GISTIC2 results. Regions with recurrent variations were selected based on a fold discovery qate *q*-value ≤0.1.

### Mutational and copy-number signature

Mutational signatures were evaluated for SBSs, IDs, and CNVs using SigProfilerMatrixGenerator (36) and SigProfilerExtractor (37) as previously described (38, 39). Signature analyses were run separately for each disease subdivided by disease status (primary, recurrent, and metastasis). Signatures were decomposed using SBS96, ID83, and CNV sets from COSMIC v.3 (RRID: SCR_002260; ref. 40).

### Oncogenic pathway analysis

Somatic variants were analyzed using the *pathways* function from the R package *maftools* (v.2.24.0; ref. 31) to verify for enrichment of the 10 canonical oncogenic signaling pathways defined by The Cancer Genome Atlas (TCGA) Pan-Cancer Analysis Project (41). The genes in the same pathway set were also used to cross-referenced amplified and deleted genes previously identified via Sequenza and GISTIC2 (32, 34).

### Inferring clonal population structure and heterogeneity

Intratumoral heterogeneity (ITH) was assessed following a previously described approach (42, 43). Tumor clonal structure was inferred using PyClone v0.13.1 (RRID: SCR_016873; ref. 44) to estimate the cellular prevalence (CP) of somatic mutations and identify distinct tumor subclones within each sample. Only samples with available tumor purity and matched normal tissue were included in the analysis. To minimize noise from singleton clusters (clusters containing only one mutation), an initial merging step was performed using average-linkage hierarchical clustering with a cutoff of 0.05, grouping clusters with similar CP and variant allele frequency (VAF). The resulting clusters were then further refined using a two-tailed Wilcoxon test, whereby pairwise comparisons of CP and VAF distributions were performed and clusters showing no significant differences in both CP and VAF (*P* > 0.05) were merged. ITH was subsequently quantified using the Shannon diversity index calculated from the CP of the final set of subclones. Specifically, the mean CP was calculated for each subclone, normalized to obtain the relative proportion of each subclone within the tumor, and the Shannon index was computed as $H^{\prime }=\;-\sum p_{i}\;\ln\left(p_{i}\right)$, capturing the diversity of subclonal composition within individual tumor samples (42, 43).

### Survival analyses

The analyses of overall survival (OSurv) were conducted using the Kaplan–Meier method with a 95% confidence interval (95% CI) and were calculated from the date of enrollment into both trials up to the date of death or last follow-up (February 2025). Patients were censored at the date of last follow-up in the absence of death. Differences between survival curves were tested through log-rank tests and Cox regression model. A Cox proportional hazard regression model was used to estimate the association between the amplification or deletion of specific cytobands for the histotype sample cohort, age (≤18 years vs. >18 years), disease stage (localized vs. metastatic at onset), sex (male vs. female), and outcome (OSurv). Significant factors (*P* < 0.05) were selected for multivariable analysis. Statistical analyses were performed using R packages *survival* v3.8.3 (Thernau T. - A Package for Survival Analysis in R. RRID: SCR_021137), *survminer* v0.4.9 (Kassambara A, Kosinski M, Biecek P. *survminer*: Drawing Survival Curves using «ggplot2». RRID: SCR_021094. Available on: https://github.com/kassambara/survminer), and *forcats* v0.5.1 [Wickham H - *forcats*: Tools for Working with Categorical Variables (Factors). Available on: https://github.com/tidyverse/forcats, https://forcats.tidyverse.org/].

## Results

### Patients and samples dataset: sequencing overview of a cohort of pediatric and AYA sarcomas

Between January 2017 and November 2024, 201 patients agreed to enroll in the pilot study SAR-GEN2016 and the national study SAR-GEN_ITA (NCT04621201). All patients underwent a biopsy or surgical resection of the tumor and healthy tissue sampling prior to starting chemotherapy, at initial diagnosis, and/or recurrence, as per clinical practice procedure. Fifty-five patients (27.4%) were excluded because of alternative diagnoses or insufficient biological material. Consequently, 146 patients were considered, for a total of 158 samples. Among these, 23 samples were excluded because of low DNA quality, whereas WXS was performed on the extracted DNA from the remaining 135 samples. Of these, 120 WXSs (85.5%) from 117 patients yielded reliable results, forming the final study cohort (Table 1; Fig. 1A). The cohort comprised 69 males (59%) and 48 females (41%), and the median age at enrollment was 14 years (range, 1–39 years). The most common tumor type was osteosarcoma, observed in 52 patients (44.5%), followed by Ewing sarcoma in 38 patients (32.5%) and RMS in 13 patients (11%; 9 embryonal RMS, 3 alveolar RMS, and 1 pleomorphic RMS). Less common tumor types included SS in four patients, (3.5%) and alveolar soft part sarcoma in two patients (1.7%). Other rare sarcomas were observed in eight patients (6.8%; Fig. 1B; Supplementary Table S1). Patients were monitored over time, with a median follow-up of 21 months (range, 1–92 months). At the last follow-up (February 2025), 68 patients (58%) were alive, whereas 49 (42%) had died. Samples were preserved as either fresh or FFPE tissue, with the majority (*n* = 107, 89%) preserved as fresh. Overall, 96 samples (80%) were obtained from the primary tumor lesion, defined as the original site of cancer initiation different from distant metastatic lesions. Sixty-eight samples (57%) were obtained at initial diagnosis. Of 120 tumors, 67 were primitive lesions (56%), 31 were recurrent tumors (26%), and 22 were metastasis (18%; Fig. 1B). Here, we limited our genomic characterization to the most prevalent histotypes (i.e., osteosarcoma, Ewing sarcoma, RMS, and SS), which together represent 91% of the entire cohort.

**Table 1.** Details of sample according to histotypes.

Tumor type	Number of samples*N* = 120 (%)	Fresh/FFPE	First diagnosis/relapse	Sampling location (primary tumor/metastasis)	Sample type (primary lesion/recurrent/metastasis)
Osteosarcoma	53 (44%)	43/10	38/15	46/7	36/11/6
Ewing sarcoma	39 (32.5%)	26/13	17/22	27/12	17/9/13
Embryonal RMS	9 (7.5%)	9/0	4/5	8/1	4/4/1
SS	5 (4.2%)	5/0	1/4	4/1	1/4/0
Alveolar RMS	3 (2.5%)	2/1	2/1	1/2	1/1/1
Liposarcoma	2 (1.7%)	2/0	1/1	2/0	1/1/0
Alveolar soft part sarcoma	2 (1.7%)	1/1	2/0	2/0	2/0/0
Pleomorphic RMS	1 (0.8%)	1/0	0/1	1/0	0/1/0
Leiomyosarcoma	1 (0.8%)	1/0	1/0	1/0	1/0/0
Primitive mesenchymal myxoid tumor	1 (0.8%)	1/0	0/1	1/0	1/0/0
Malignant fibrous histiocytoma of bone	1 (0.8%)	1/0	1/0	1/0	1/0/0
Epithelioid sarcoma	1 (0.8%)	1/0	0/1	0/1	0/0/1
Infantile fibrosarcoma	1 (0.8%)	1/0	1/0	1/0	1/0/0
Dedifferentiated liposarcoma	1 (0.8%)	1/0	1/0	1/0	1/0/0

### Characterization of the acquired mutational landscape revealed histotype-specific mutations within a context of low tumor mutational burden and childhood cancer–associated signature

**Figure 2.**
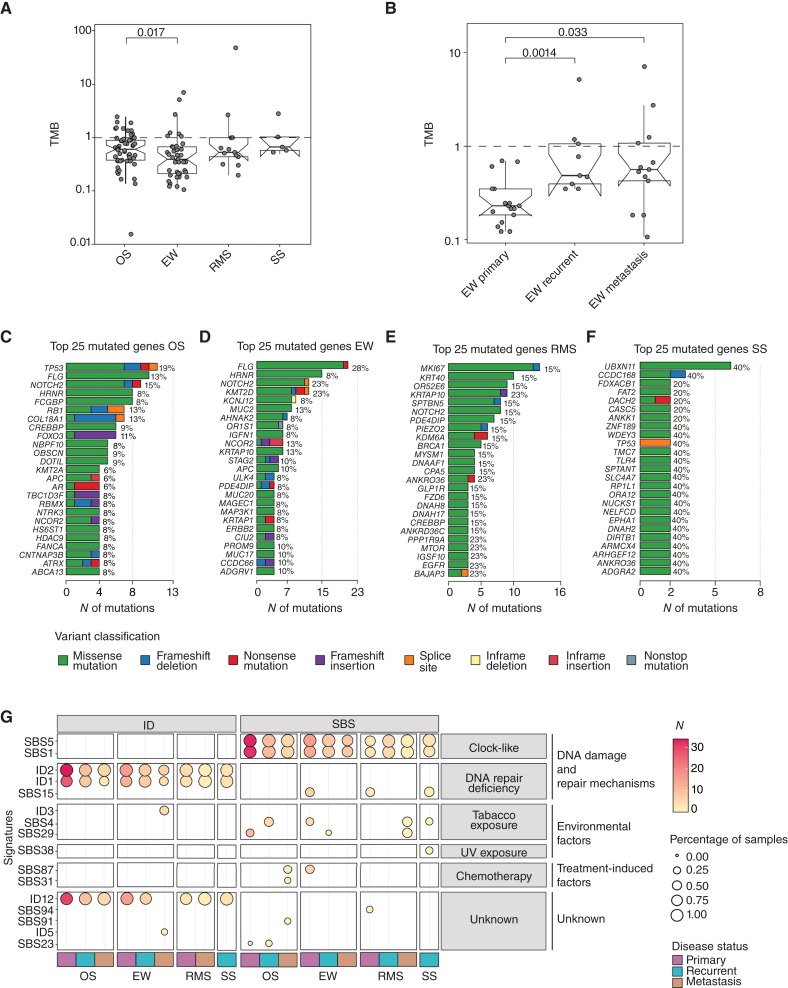
Genomic landscape of the four sarcoma subtypes. **A** and **B,** Box plots show the distribution of TMB on the *y*-axis, in which each dot represents an individual tumor sample. The horizontal dotted line at 1 mutation per megabase (mut/Mb) denotes the threshold used to separate lowly and highly mutated tumors. **A,** TMB distribution across all tumor samples of osteosarcoma (OS), Ewing sarcoma (EW), RMS, and SS. Statistically significant difference was observed between OS and EW samples (two-tailed Wilcoxon test). **B,** TMB distribution stratified by disease status (primary, recurrent, and metastasis) within the EW cohort. Significant differences were observed between primary and recurrent tumors (two-tailed Wilcoxon test) and between primary and metastatic tumors (two-tailed Wilcoxon test). **C–F,** Bar plots summarizing the top 25 mutated genes for each sarcoma subtypes, based on all tumor samples and without stratification by disease status. Genes are listed on the left side of each plot, with the corresponding percentage of mutated samples shown on the right. The percentages represent the number of unique samples harboring a mutation divided by the total number of samples in each sarcoma subtype (OS = 53, EW = 39, RMS = 13, and SS = 5). The *x*-axis shows the total number of mutations identified per gene. Color code represents the type of variants. **G,** Summary of the SBS and small indel signatures, stratified by disease status for each tumor subtype. On the left, the signature names. Each dot represents a mutational signature. The color of the dot (N) indicates the number of samples in which the signature is present, whereas the dot size represents the percentage of samples carrying that signature within that cohort. Signatures are grouped horizontally based on their classification and vertically by disease. The color bar at the bottom indicates the disease status.

To assess the landscape of genomic alterations in the four sarcoma histotypes, we identified somatic SBSs and indels from WXS data (Fig. 2). First, we evaluated the tumor mutational burden (TMB) defined as the frequency of nonsilent somatic mutations in the sequence d exome. TMB was initially calculated using all tumor samples within each tumor subtype and subsequently analyzed according to disease status. The median value across the four sarcoma histotypes was 0.54 muts/Mb (Fig. 2A; Supplementary Table S1). Three Ewing sarcomas, one embryonal RMS, one osteosarcoma, and one SS were classified as “pediatric high” (TMB ranging between 2 and 10 muts/Mb), and one pleomorphic RMS as “hypermutator” sample (>10 muts/Mb), according to the classification of childhood cancers proposed by Gröbner and colleagues (Supplementary Table S1; ref. 45). Interestingly, Ewing sarcomas showed a lower TMB compared with osteosarcoma samples (Ewing sarcoma median TMB = 0.39 muts/Mb vs. osteosarcoma median TMB = 0.60 muts/Mb, *P* = 0.017, two-tailed Wilcoxon test; Fig. 2A). Furthermore, the median TMB (0.22 muts/Mb) of Ewing sarcomas derived from treatment-naïve primary tumors was lower compared with refractory tumors and metastatic lesions (median TMB value of Ewing sarcoma recurrent tumors = 0.48 muts/Mb, *P* = 0.001, two-tailed Wilcoxon test - median TMB value of Ewing sarcoma metastases = 0.56 muts/Mb, *P* = 0.03, two-tailed Wilcoxon test; Fig. 2B). No other significant differences were observed in the whole cohort. We next evaluated the mutational profile of each sarcoma cohort using all available tumor samples, without stratification by disease status, to uncover potential recurrent alterations shared among samples. Within the osteosarcoma cohort, the most frequently mutated genes were *TP53* (19%), *NOTCH2* (15%), *RB1*, *COL18A1*, and *FLG* (each 13%; Fig. 2C). In the Ewing sarcoma cohort, *FLG* is the most frequently mutated gene (28% of samples), followed by *KMT2D* and *NOTCH2* (each 23%; Fig. 2D). In the RMS group, the most frequently altered genes were *ANKRD36*, *EGFR*, *BAIAP3*, *IGSF10*, *KRTAP10*, *MTOR*, and *PPP1R9A*, observed in 23% of the samples (Fig. 2E), and two independent patients (one alveolar RMS and one embryonal RMS) harbored an identical NRAS missense mutation (c.C181A, p.Q61K). Any differences were detected among RMS subtypes because of the low number of samples in this specific disease cohort. In the SS cohort, the most recurrently mutated genes are summarized in Fig. 2F. Two of five SS samples (40%) shared a common *TP53* splicing mutation (c.560-1G>A).

To better characterize our samples, we evaluated the signatures for SBSs and small indels, stratified by disease status (Fig. 2G). The mutational patterns of the entire cohort were recapitulated by the known COSMIC database (46). SBS1 and SBS5 were the most prevalent signatures detected across the cohort. Both signatures have been recurrently reported in pediatric cancers, including pediatric sarcoma (42). SBS1, characterized by C > T transitions at methylated CpG dinucleotides arising from spontaneous deamination of 5-methylcytosine, and SBS5, a clock-like signature of unknown etiology (39, 47). The ID signatures showed a more homogeneous distribution among the different histotypes. Three COSMIC ID signatures (ID1, ID2, and ID12) were identified in common between osteosarcoma, Ewing sarcoma, RMS, and SS samples. ID1 and ID2, which correlate with slippage during DNA replication of the replicated DNA strand, were present in most samples. Similarly to SBS1, ID1 and ID2 have been recurrently found in pediatric cancers (39) and were associated with SBS1 in nonhypermutated samples. Although ID12 has been previously identified in pediatric patients with brain tumors (48), its etiology is unknown.

### CNV analysis highlighted a high CNV burden in osteosarcoma and tetraploidy-associated signatures in translocation-negative sarcomas

**Figure 3.**
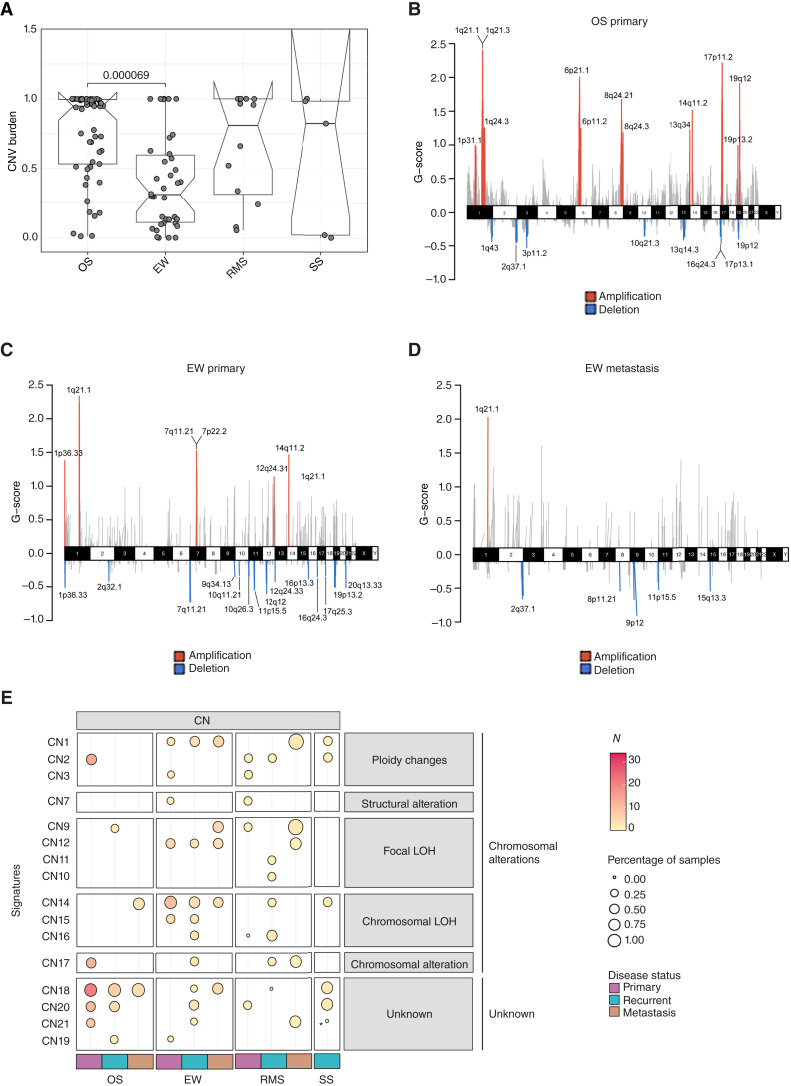
CNV across sarcoma subtypes. **A,** CNV burden is shown with a box plot highlighting significant differences across all tumor samples from osteosarcoma (OS), Ewing sarcoma (EW), RMS, and SS, without stratification by disease status. A statistically significant difference was observed between EW and OS samples (two-tailed Wilcoxon test). **B–D,** Recurrent and significant CNVs were identified in OS and EW samples using GISTIC2 for primary (**B** and **C**) and metastatic (**D**) tumors. The cytobands are visualized using the R package *maftools*. Blue bars indicate deletions, red bars indicate amplifications, and gray bars represent nonsignificant CNVs. The G-score represents the amplitude and frequency of the CNVs across tumors of interest. **E,** Distribution of CN signatures across the cohorts, stratified by disease status for each tumor subtype. Left, signature names. Each dot represents a mutational signature. The color of the dot indicates the number (N) of samples harboring the signature, whereas the size of the dot reflects the percentage of samples carrying that signature. Signatures are grouped horizontally based on their classification and vertically by disease. The color bar at the bottom indicates the disease status.

To further investigate the genomic complexity of sarcomas, we subsequently analyzed CNVs and their associated signatures. The CNV burden, defined as the fraction of genome with copy-number alteration on the size of the total genome, was calculated using *sigminer* (34) for all tumor samples in each sarcoma subtype. The median CNV burden value of the overall cohort was 0.64 (Fig. 3A). As expected based on previous genomic studies, osteosarcoma samples exhibited a significantly higher CNV burden compared with Ewing sarcoma (median Ewing sarcoma CNV burden value of 0.31 vs. median osteosarcoma CNV burden value = 0.95, *P* < 0.001, two-tailed Wilcoxon test), reflecting the well-established chromosomal instability that characterizes genomically complex sarcomas (Fig. 3A).

We identified genomic regions and genes significantly amplified or deleted using GISTIC2 (26) and assessed their recurrence across primary, recurrent, and metastatic tumors for each histotype. We found significant (q-value ≤ 0.1) cytobands in osteosarcoma (primary and recurrent) and Ewing sarcoma (primary, recurrent, and metastasis) samples (Fig. 3B–D; Supplementary Table S3). The cytoband 6p21.1 was commonly amplified among the osteosarcoma primary and recurrent tumors, containing *VEGFA* and *RUNX2*. The latter is a known gene related to osteosarcoma (48, 49), and it was reported to regulate *VEGFA* expression, thereby linking osteogenic differentiation signals to angiogenic pathways. In primary osteosarcoma samples, the most frequently deleted regions were 13q14.3 (78% of samples) and 17p13.1 (61% of samples), which include the *RB1* and *TP53* genes, respectively (Fig. 3B). The loss of these genes is a known oncogenic event in osteosarcoma and reflects the genomic complexity feature of this tumor type (48). Meanwhile, in the Ewing sarcoma cohort, the most frequently amplified cytoband was 1q21.1, observed in 71% of primary tumors and 58% of metastases (Fig. 3C and D). This chromosomal region included the *FAM72D* gene, a candidate driver whose amplification has been reported in several cancer types (50).

Next, we analyzed the distribution of copy-number signatures across our cohort, with samples stratified by disease status. At least one of the ploidy-associated signatures (CN1, CN2, and CN3) was detected in most samples (Fig. 3E), underscoring the widespread occurrence of large-scale chromosomal changes in sarcomas. The CN1 signature, typically associated with diploidy, was predominantly observed in Ewing sarcoma and SS, both tumor types characterized by balanced genomes and by the presence of a well-established recurrent driver gene fusion. In contrast, the CN2 signature, indicative of tetraploidy and often linked to whole-genome duplication events, was most frequently found in osteosarcoma and RMS samples (Fig. 3E). It is worthy of note that tumors in our cohort enriched with CN2 did not harbor known gene fusions. This suggested that the tetraploid associated signature was related to genomic complex sarcomas instead of fusion-driven sarcomas in the present study.

### ITH differed between bone and soft-tissue osteosarcoma samples and across different disease status in Ewing sarcoma

We assessed subclonal heterogeneity for each tumor sample in our cohort by calculating ITH scores using the Shannon diversity index derived from the mean cluster CP estimated with PyClone (44), as described in “Materials and Methods”. ITH scores were obtained for 49 osteosarcoma, 38 Ewing sarcoma, 12 RMS, and 5 SS samples and were first compared to assess differences between sarcoma subtypes. ITH values were subsequently stratified according to disease status and tumor location. Overall, no significant differences in ITH were observed among RMS and SS samples. In contrast, osteosarcoma and Ewing sarcoma exhibited meaningful differences in ITH, and subsequent analyses therefore focused on these two subtypes to further explore subclonal heterogeneity.

**Figure 4.**
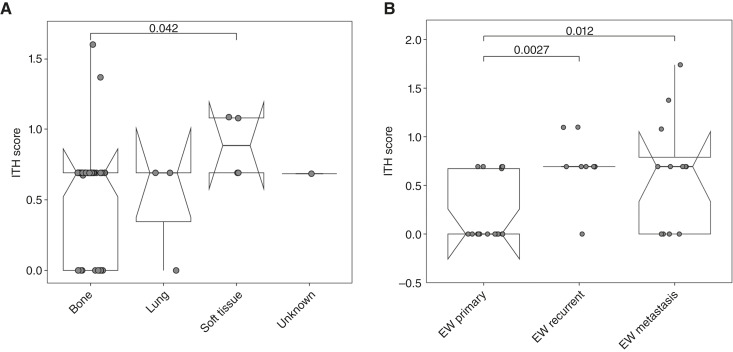
Assessment of ITH using the Shannon diversity index score. Box plots show the distribution of ITH scores (*y*-axis), in which each dot corresponds to an individual tumor sample. The ITH scores were assessed across sarcoma subtypes, disease status, and tumor location, with meaningful differences observed in osteosarcoma (OS) and Ewing sarcoma (EW) but not in RMS or SS. ITH distribution stratified by (**A**) tumor location in OSs and (**B**) disease status in EWs.

We found osteosarcoma samples located in bone to be significantly different from those arising in soft tissue (two-tailed Wilcoxon test, *P* = 0.042), regardless of the disease status (Fig. 4A). Furthermore, our data showed that recurrent and metastatic Ewing sarcomas were significantly more heterogeneous than treatment-naïve primary Ewing sarcoma samples (two-tailed Wilcoxon test, *P* = 0.0027 and *P* = 0.012, respectively), consistent with the increased TMB reported above (Fig. 4B). These findings suggested the hypothesis that in Ewing sarcoma samples, there was an accumulation of mutations, correlated with an increase of subclonal populations in response to therapy, as already reported in other solid tumors, potentially contributing to therapeutic resistance (44).

### Oncogenic pathway dysregulation in pediatric and AYA sarcomas revealed RTK–RAS and NOTCH as consistently altered pathways across subtypes

**Figure 5.**
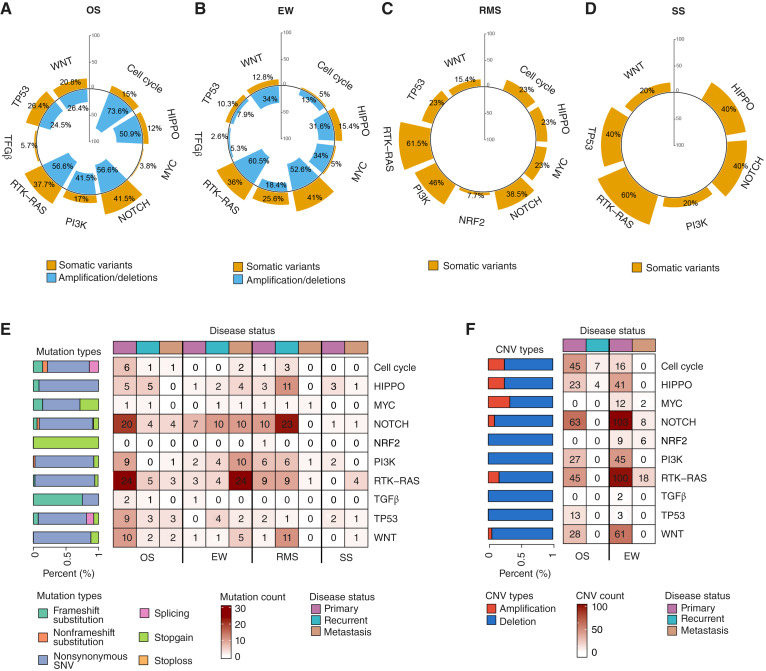
Oncogenic signaling pathways. **A–D,** Mirrored circular bar plots depict the overall enrichment of oncogenic pathways in osteosarcoma (OS; **A**), Ewing sarcoma (EW; **B**), RMS (**C**), and SS (**D**). Each bar represents a single pathway, with the height indicating the proportion of samples harboring corresponding somatic alterations. Somatic variants are shown in yellow and CNVs in light blue, with the corresponding percentage of affected samples. **E** and **F,** The heatmaps provide a detailed view of (**E**) variants and (**F**) CNVs in genes of each pathway stratified by disease status and, grouped by disease subtype. Top color bar indicates disease status. Bar plots on the right represent the proportion of each alteration type across pathways, cells, and corresponding color code.

To better understand the biological impact of the genomic alterations identified in our cohort, we investigated whether the somatic SBSs, indels, and CNVs were part of the 10 canonical oncogenic signaling pathways defined by the The Cancer Genome Atlas Pan-Cancer Analysis Project (41). Although each sarcoma subtype and disease status displayed its own distinct pattern of pathway dysregulation, RTK–RAS and NOTCH emerged as the most frequently altered pathways across the four major sarcoma subtypes (Fig. 5; Supplementary Table S4).

In detail, we observed that in the osteosarcoma cohort the RTK–RAS and NOTCH pathways were most frequently altered, with CNVs in 56.6% of samples and somatic mutations in 37.7% and 41.5% of samples, respectively (Fig. 5A). The NOTCH pathway disruption was consistently observed between primary, recurrence and metastatic samples, with frequent involvement of *NOTCH2/3*. Moreover, cell cycle alterations were particularly found in osteosarcoma, with CNVs in 73.6% of samples and additional mutations in 15% of samples, with *RB1* frequently deleted and *CCNE1* amplified, as also described elsewhere (10). p53 pathway disruption was frequent across disease status, in contrast with the other sarcoma subtypes and consistent with its known involvement in osteosarcoma pathogenesis (51).

In Ewing sarcoma, mutations in RTK–RAS and NOTCH were detected in 36% and 41% of cases, respectively, with CNVs exceeding 50% (Fig. 5B). Primary tumors showed deletions in regulators such as *HRAS*, *NF1*, *MAPK3*, and *CBL*, whereas recurrent and metastatic cases were enriched in clustered lesions affecting *EGFR*, *ERBB2/3/4*, *FGFR1/3/4*, *IGF1R*, *MET*, *PDGFRA/B*, *RET*, and *ROS1*. The PI3K and Hippo pathways were also frequently altered by CNVs in Ewing sarcoma, although with lower mutation frequencies (18.4% and 31.6%). Alterations in the cell cycle were more common in recurrent and metastatic stages, whereas *TP53* alterations were rare.

The RMS and SS cohorts, despite their smaller sample sizes, also showed enrichment of the RTK–RAS pathway. Specifically, within the RMS cohort, two independent patients harbored an identical *NRAS* missense mutation (c.C181A, p.Q61K). The latter is part of the RTK–RAS pathway, which is the most mutated pathway in this sarcoma histotype (52). RMS recurrent tumors were characterized by *NRAS* mutations and lesions in RTKs (*EGFR*, *ERBB2/4*, *IGF1R*, and *NTRK2/3*) and RAS regulators (*IRS1*, *NF1*, and *RASA1*; Fig. 5C and D). SS exhibited fewer events but included alterations in *MET*, *NTRK3*, *RASGRF1*, and *SCRIB*. Secondary pathways, including PI3K, Hippo, and cell cycle, showed sporadic mutational involvement across these subtypes (Fig. 5E and F). Taken together, these results suggested that recurrent alterations in RTK–RAS and NOTCH signaling, along with cell cycle and p53 disruption, are central to sarcoma pathogenesis and vary according to disease subtype and progression.

### CNVs and correlation with overall survival in osteosarcoma cohort

To evaluate the potential impact of somatically acquired alterations on the outcome of patients in the four sarcoma cohorts, we conducted an overall analysis to estimate the association between the amplification or deletion of specific cytobands identified by GISTIC2 for disease subtype, age, stage, sex and outcome. We found significant results only in the osteosarcoma primary cohort, using both univariate and multivariate survival analyses. Among the cytobands described above, only 10q21.3 in primary osteosarcoma, present in 64% of samples, showed a significant correlation with patient survival (Supplementary Fig. S1). We found that patients with the deletion of 10q21.3 displayed superior survival rates, with an OSurv of 86% at 12 months and 70% at 24 months, compared to 53% and 30%, respectively, in patients without the deletion. This difference was statistically significant both in univariate analysis (Log-rank *P* = 0.015) and multivariate analysis (HR = 0.04; 95% CI, 0.0005–0.29; *P* = 0.015).

### A precision medicine approach through the integration of genomic data

To facilitate therapeutic approaches tailored to the cancer molecular profiles, the genomic aberrations identified were discussed by a MTB. In 71 of 120 analyzed samples (59%), at least one potentially actionable genomic alteration was identified according to the criteria previously defined in the European MAPPYACTS study (53) and according to ESCAT classification (23). Among the 279 “potentially actionable” findings, 12 were SBSs (10 somatic and 2 germline), 263 were focal CNVs, including 230 amplifications/high-level gains and 33 deletions, and 4 were cases of elevated TMB. All findings were classified as ESCAT tier II or III. Among the 71 cases harboring at least one potentially actionable genomic alteration, 56% (*n* = 40) of tumor samples were collected at initial diagnosis and 31 were collected at relapse. Cases enrolled at initial diagnosis were not evaluated for targeted therapy, as standard first-line chemotherapy was promptly initiated. In contrast, samples obtained at relapse, were considered for targeted treatments due to the absence of effective standard therapy options. Of these, 5 of 31 patients (16%) received a matched targeted therapy based on the molecular profiling results (Table 2). The remaining 26 patients did not receive target therapy because (i) they were in complete remission after second- or third-line standard treatment, (ii) the rapid disease progression precluded further therapeutic interventions, or (iii) the targeted agent was neither available in the pediatric setting nor accessible in clinical trials.

**Table 2.** Five patients received a matched targeted therapy based on the molecular profiling results.

​	Disease	Disease status	Age (years)	Alteration type	Gene	Matched treatment	Number of treatment lines before matched treatment	Outcome
Case 1	Alveolar soft part sarcoma	Diagnosis	15	High TMB	—	Immunotherapy phase I trial at relapse	3	Radiologic stable disease after two cycles and then radiologic progressive disease
Case 2	Epitheliod sarcoma	Relapse	15	Deletion	*SMARCB1*	EZH2 inhibitor (compassionate use)	4	Radiologic progressive disease
Case 3	Ewing sarcoma	Relapse	12	High TMB	—	Immunotherapy phase I trial	4	Radiologic progressive disease
Case 4	Ewing sarcoma	Relapse	16	Gene fusion	*EWSR1::FLI1*	Genomic inclusion criteria for a phase I trial with PARP inhibitor + ATM/ATR inhibitor	3	Radiologic progressive disease after two cycles
Case 4	Ewing sarcoma	Relapse	16	Amplification	*FGFR4*	Genomic inclusion criteria for a phase I trial with multitarget tyrosine kinase inhibitor	4	Radiologic stable disease after two cycles and then radiologic progressive disease
Case 5	Ewing sarcoma	Relapse	19	Mutation germline	*PALB2*	Temozolomide + PARP inhibitor (off-label use)	5	Radiologic progressive disease; however, the patient had clinical benefit during the first cycle with pain relief

## Discussion

The SAR-GEN-2016 and SAR-GEN_ITA clinical trials represented the first national initiative to outline a comprehensive genomic profiling analysis specifically dedicated to pediatric and AYA patients with bone and soft-tissue sarcomas within the Italian network of the AIEOP. The aims were to provide an extensive molecular characterization of these rare and aggressive malignancies, uncovering both shared and subtype-specific genomic alterations.

We performed WXS on 120 bone and soft-tissue sarcoma samples, utilizing matched tumor and germline (blood) DNA. This approach enabled a more accurate analysis, as recently demonstrated (45) compared with tumor-only sequencing, while still encompassing all relevant genomic alterations for targeted therapy decision and for discovery approach. Our findings align with the established genomic frameworks of pediatric and AYA sarcomas (11, 54, 55). Specifically, our cohort displayed a lower TMB than adult sarcomas, with only a minority of samples classified as “pediatric high” and even fewer exhibiting hypermutation (45). The role of the TMB has emerged to be predictive in immunotherapy response and has also been described as a prognostic factor in solid cancers, including RMS (56). In the Ewing sarcoma cohort, TMB was significantly higher in metastasis than in primary tumors. TMB was also higher in recurrent previously treated samples than in treatment-naïve cases, consistent with observations in other solid tumors and potentially linked to chemoresistance (57).

In Ewing sarcomas the presence of a more subclonal population is associated with more aggressive disease, as has been observed in other cancer types (58, 59). Although we did not systematically analyze samples from the same patient, our results showed the accumulation of mutations and the gain of subclonal population in Ewing sarcomas during tumor progression. These findings indicate that ITH increased with disease progression and it might potentially contribute to therapeutic resistance, as high ITH has been shown in other tumors to enable rapid adaptation through selection of resistant subclones, promote metastasis, and correlate with poorer outcomes (42, 60). Tumor location also appears to influence ITH. In our osteosarcoma cohort, samples originating in bone showed significantly different ITH scores compared with those arising in soft tissue, reflecting the spatially distinct subclonal architecture that has been reported in other cancers (57). We focused our analysis on osteosarcoma and Ewing sarcoma, as differences in ITH for RMS and SS were not significant. However, a limitation of our study is that we were unable to account for potential confounding factors such as biopsy size, as this information was not available. The aim of our analysis was not to reconstruct clonal evolution, as longitudinal or multi-region samples from the same tumors were not available and dedicated approaches exist for this purpose (61). Instead, ITH was used as a summary measure to characterize subclonal diversity within individual tumor samples. The Shannon diversity index, used here to quantify ITH, has been widely applied in other studies (42) to infer subclonal diversity from VAF. Although informative, ITH does not capture evolutionary relationships between clones, may underestimate rare subclones, and can be influenced by tumor purity or CNVs. Despite these limitations, our findings are consistent with prior work showing that higher ITH reflects increased evolutionary potential and can inform understanding of disease progression and therapy resistance (41).

Sarcomas typically have a low somatic mutation burden and are instead shaped by karyotypic instability, with CNVs exceeding point mutations (38). Consistent with large-scale studies such as TCGA, mutational landscapes are dominated by the clock-like SBS1 and SBS5 signatures, which are generally considered as background mutational processes rather than sarcoma-specific drivers (62). We observed the same pattern in our cohort, with SBS1 and SBS5 as the most prevalent signatures. We also found that a substantial contribution from CN signatures linked to chromosomal instability and loss of heterozygosity (LOH) in our cohort, including CN9 (diploid CIN), CN10 to CN12 (LOH with whole-genome duplication), CN 13 to CN16 (chromosomal LOH), and CN17 (homologous recombination deficiency), with CN17-attributed tumors showing recurrent LOH affecting key tumor-suppressors such as *CDKN2A*, *RB1*, and *TP53* (38). Our osteosarcoma cohort exhibited a high CNV burden, with enrichment of the CN2 signature, indicative of a highly complex genome compared with the translocation-associated sarcomas (38). Interestingly, all samples in our cohort enriched with CN2 (osteosarcoma, RMS, and one SS) did not harbor known gene fusions, highlighting that the tetraploid-associated signature was related to genomic complex sarcomas instead of fusion-driven sarcomas in the present study.

Signaling pathways exhibited different somatic alterations across tumors, highlighting complex biological interactions crucial for therapy development and patient care. RTK–RAS and NOTCH pathways emerged as the most consistently altered across all sarcoma types. Preclinical studies have shown that NOTCH signaling is associated with prognosis in osteosarcoma, and investigations of the NOTCH inhibitors such as CB-103 and RO4929097 have been conducted in solid tumors showing preliminary evidence of clinical antitumor activity (63, 64). In addition, osteosarcoma samples showed dysregulation of the p53 and cell-cycle pathways (51). Recently, clinical activity of palbociclib, a *CDK4/6* inhibitor, in osteosarcoma was anecdotally reported (65). However, studies in combination with other therapies such as regorafenib or bromodomain inhibitors should be explored, especially in patients with a concomitant *TP53* alteration, which seemed to be strongly related to *CDK4/6* inhibitor resistance (18). Pathway-level analysis can be useful to redefine precision medicine approaches by targeting all the signaling networks, especially for those tumors without pathognomonic driver targets. This strategy provides a strong rationale for informing both ongoing and future precision oncology clinical trials (18).

A major unmet clinical need in sarcoma, particularly in osteosarcoma, is the identification of robust prognostic biomarkers to enable more precise therapeutic stratification (66). We therefore investigated the clinical impact of somatic genomic alterations across four sarcoma cohorts. However, interpretable signals emerged only in the osteosarcoma primary cohort, which was the largest. In this cohort, loss of chromosome 10q21.3 was associated with improved OSurv. Although exploratory, this finding supports further validation in larger independent cohorts and prospective studies to define its clinical relevance and potential utility.

More broadly, integrating genomic data for discovery purposes with clinical tumor molecular profiles has considerable potential to advance precision medicine. Nevertheless, substantial challenges remain in translating these approaches into routine clinical pediatric practice, particularly for bone and soft-tissue sarcomas (17).

Here, we described that the 59% of the analyzed samples showed at least one potentially actionable genomic alteration, although none of them were classified as ESCAT tier I (23), and five patients ultimately received a matched targeted therapy based on the molecular results. The proportion of patients who ultimately benefited from targeted therapy is lower than in other trials reported in the literature (53, 67). This discrepancy is primarily attributable to our enrollment criteria, which included patients at initial diagnosis or first relapse who had achieved a stable complete remission following first- or second-line standard treatments and they did not need any further treatment. Among the relapsed or refractory patients, only a low proportion was treated with a matched drug. The limited translation of genomic findings into matched therapies observed in our cohort likely reflects both the unique biological features of sarcomas, especially osteosarcoma and Ewing sarcoma, which are known to be characterized by a low mutational burden (63), and the presence of systemic barriers. These include restricted access to genotype-matched clinical trials for children and AYA (68), the limited availability of approved targeted agents for rare alterations, timing of testing late in the disease course, and regulatory or reimbursement constraints. In this regard, a recent Italian consensus proposed that evidence classified as ESCAT tier II or III should be considered sufficient for patients with sarcoma, given the rarity of many histotypes and molecular alterations encountered (69). Although pediatric genomic oncology programs are feasible and remain crucial for fostering translational research for rare cancers with significant unmet needs, the real-world clinical benefit of current DNA-based profiling in pediatric and AYA sarcomas is still limited. Further work is therefore needed to evaluate complementary approaches, such as the incorporation of liquid biopsy or single-cell transcriptomic, to determine whether they can add meaningful value to precision oncology in this setting (17, 70–73).

In summary, this study provides a characterization of pediatric and AYA sarcomas. Despite their rarity, there is broad consensus within the scientific and clinical communities on the strategic value of systematically collecting and sharing biological samples from these tumors to accelerate translational research (74). Such efforts are critical not only for improving clinical management, through the identification of prognostic biomarkers and novel therapeutic targets, but also for advancing our fundamental understanding of sarcoma biology and pathogenesis. Moving forward, the integration of early genomic testing, routine implementation of MTBs, broader access to targeted therapies, and promotion of data sharing to increase cohort sizes and strengthen statistical power will serve as key pillars in the effort to improve outcomes for pediatric and AYA patients with sarcoma.

## Supplementary Material

Supplementary Table 1 Clinical and sample overview of Sargen and Sargen-ITA patients

Supplementary Table 2 ESMO Scale for Clinical Actionability of molecular Targets (ESCAT)

Supplementary Table 3 Significant cytobands identified in osteosarcoma (primary and recurrent) and Ewing’s sarcoma (primary, recurrent, metastasis) with the respective genes

Supplementary Table 4 Genes detected to be involved in oncogenic pathways

Supplementary Figure 1 Supplementary Figure 1

## Data Availability

The data are deposited in the Sequence Read Archive repository, accession number PRJNA1369170. The original code has been archived and is publicly accessible at Code Ocean at https://codeocean.com/capsule/2018854/tree/v1 and GitHub at https://github.com/ceredamatteo-lab/Tirtei_et_al-SARGEN. Additional details necessary to reproduce the analyses described in this study are available from the correspondent contact upon request.
