# Supplementary material for: Mapping Genomic Heterogeneity in Pediatric and Adolescent–Young Adult Sarcomas: Insights from the Italian SAR-GEN2016 and SAR-GEN_ITA Prospective Multicenter Trials
Source: Cancer Res Commun. 2026 Apr 17;6(4):857–72. doi: 10.1158/2767-9764.CRC-25-0697 (PMC13090861; doi:10.1158/2767-9764.CRC-25-0697)
Supplement: Supplementary Table 2 — ESMO Scale for Clinical Actionability of molecular Targets (ESCAT) [file crc-25-0697_supplementary_table_2_suppst2.pdf]

Supplementary Table 2: ESMO Scale for Clinical Actionability of molecular Targets (ESCAT) [1]

	ESCAT evidence tier	Required level of evidence	Clinical value class	Clinical implication
**Ready for routine use**	I: Alteration-drug match is associated with improved outcome in clinical trials	I-A: prospective, randomised clinical trials show the alteration-drug match in a specific tumour type results in a clinically meaningful improvement of a survival end point.<br><br>I-B: prospective, non-randomised clinical trials show that the alteration-drug match in a specific tumour type, results in clinically meaningful benefit as defined by ESMO MCBS 1.1<br><br>I-C: clinical trials across tumour types or basket clinical trials show clinical benefit associated with the alteration-drug match, with similar benefit observed across tumour types	Drug administered to patients with the specific molecular alteration has led to improved clinical outcome in prospective clinical trial(s)	Access to the treatment should be considered standard of care
**Investigational**	II: alteration-drug match is associated with antitumour activity, but magnitude of benefit is unknown	II-A: retrospective studies show patients with the specific alteration in a specific tumour type experience clinically meaningful benefit with matched drug compared with alteration-negative patients.<br><br>II-B: prospective clinical trial(s) show the alteration-drug match in a specific tumour type results in increased responsiveness when treated with a matched drug, however, no data currently available on survival end points	Drug administered to a molecularly defined patient population is likely to result in clinical benefit in a given tumour type, but additional data are needed	Treatment to be considered 'preferable' in the context of evidence collection either as a prospective registry or as a prospective clinical trial
**Hypothetical target**	III: alteration-drug match suspected to improve outcome based on clinical trial data in other tumour type(s) or with similar molecular alteration	III-A: clinical benefit demonstrated in patients with the specific alteration (as tiers I and II above) but in a different tumour type. Limited/absence of clinical evidence available for the patient-specific cancer type or broadly across cancer types.<br><br>III-B: an alteration that has a similar predicted functional impact as an already studied tier I abnormality in the same gene or pathway, but does not have associated supportive clinical data	Drug previously shown to benefit the molecularly defined subset in another tumour type (or with a different mutation in the same gene), efficacy therefore is anticipated for but not proved	Clinical trials to be discussed with patients
	IV: pre-clinical evidence of actionability	IV-A: evidence that the alteration or a functionally similar alteration influences drug sensitivity in preclinical in vitro or in vivo models.<br><br>IV-B: actionability predicted in silico	Actionability is predicted based on preclinical studies, no conclusive clinical data available	Treatment should 'only be considered' in the context of early clinical trials. Lack of clinical data should be stressed to patients
**Combination development**	V: alteration-drug match is associated with objective response, but without clinically meaningful benefit	Prospective studies show that targeted therapy is associated with objective responses, but this does not lead to improved outcome	Drug is active but does not prolong PFS or OS, probably in part due to mechanisms of adaptation	Clinical trials assessing drug combination strategies could be considered
	X: lack of evidence for actionability	No evidence that the genomic alteration is therapeutically actionable	There is no evidence, clinical or preclinical, that a genomic alteration is a potential therapeutic target	The finding should not be taken into account for clinical decision

Reference

1. J. Mateo, D. Chakravarty, R. Dienstmann, S. Jezdic, A. Gonzalez-Perez, N. Lopez-Bigas et al. A framework to rank genomic alterations as targets
