# Supplementary figures and images for: Mapping Genomic Heterogeneity in Pediatric and Adolescent–Young Adult Sarcomas: Insights from the Italian SAR-GEN2016 and SAR-GEN_ITA Prospective Multicenter Trials
Source: Cancer Res Commun. 2026 Apr 17;6(4):857–72. doi: 10.1158/2767-9764.CRC-25-0697 (PMC13090861; doi:10.1158/2767-9764.CRC-25-0697)

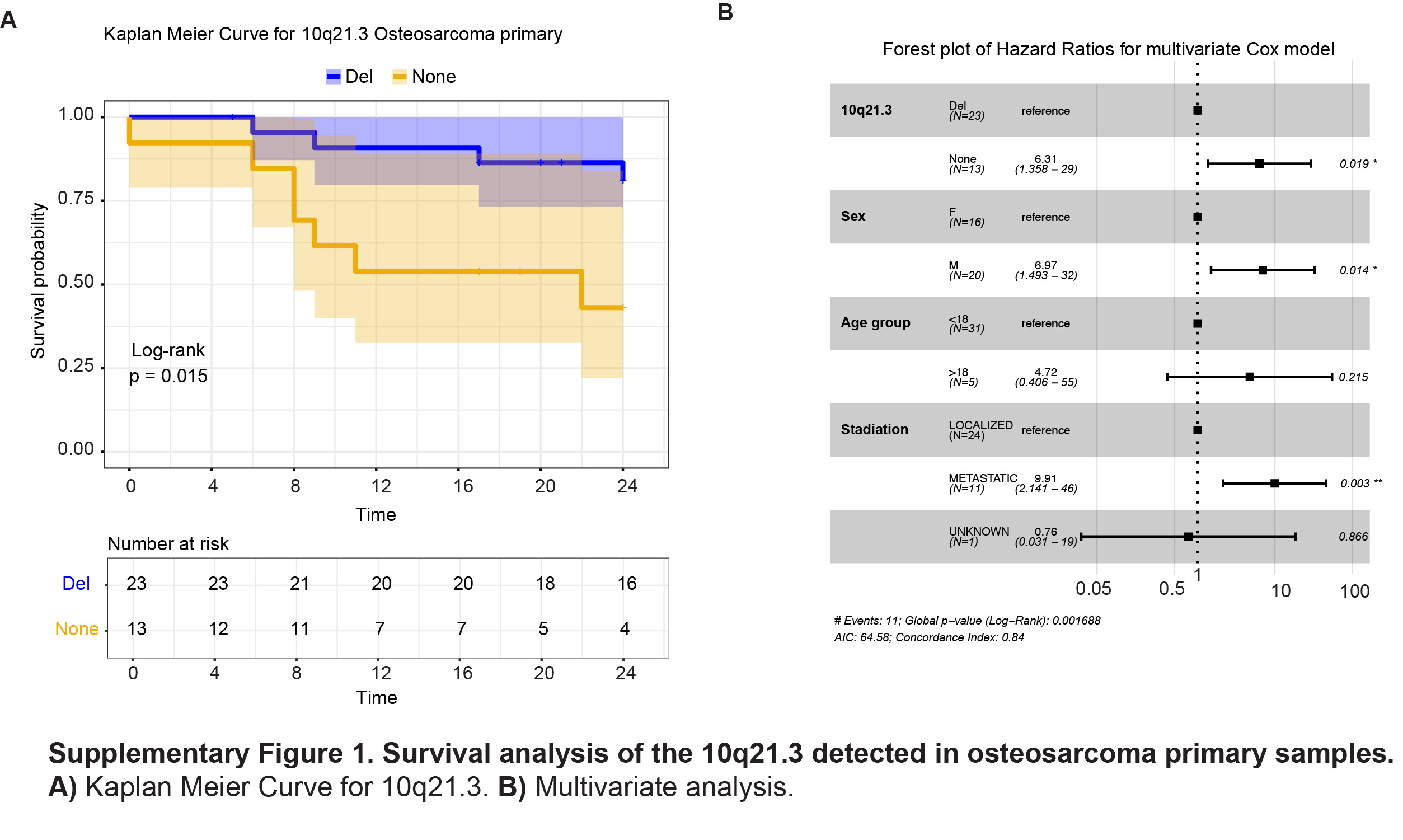

Supplement: Supplementary Figure 1 [file crc-25-0697_supplementary_figure_1.png]
